# Sex differences in the association between cardiovascular diseases and dementia subtypes: a prospective analysis of 464,616 UK Biobank participants

**DOI:** 10.1186/s13293-022-00431-5

**Published:** 2022-05-07

**Authors:** Caiyun Dong, Chunmiao Zhou, Chunying Fu, Wenting Hao, Akihiko Ozaki, Nipun Shrestha, Salim S. Virani, Shiva Raj Mishra, Dongshan Zhu

**Affiliations:** 1 Centre for Health Management and Policy Research, School of Public Health, Cheeloo College of Medicine, Shandong University, Jinan, 250012 China; 2 NHC Key Lab of Health Economics and Policy Research, Shandong University, Jinan, 250012 China; 3 Department of Breast Surgery, Jyoban Hospital of Tokiwa Foundation, Iwaki, Fukushima Japan; 4 Department of Gastrointestinal Tract Surgery, Fukushima Medical University, Fukushima, Japan; 5 Department of Primary Care and Mental Health, University of Liverpool, Liverpool, UK; 6 Michael E. DeBakey VA Medical Center and Baylor College of Medicine, Houston, TX USA; 7 Academy for Data Sciences and Global Health, Kathmandu, Nepal; 8 Melbourne School of Population and Global Health, The University of Melbourne, Melbourne, VIC Australia; 9 Department of Epidemiology, School of Public Health, Cheeloo College of Medicine, Shandong University, 44 Wenhuaxi Road, Jinan, 250012 Shandong China

**Keywords:** Sex difference, Coronary heart diseases, Stroke, Heart failure, Alzheimer’s disease, Vascular dementia, Cohort study

## Abstract

**Background:**

Whether the association of cardiovascular diseases (CVDs) with dementia differs by sex remains unclear, and the role of socioeconomic, lifestyle, genetic, and medical factors in their association is unknown.

**Methods:**

We used data from the UK Biobank, a population-based cohort study of 502,649 individuals. We used Cox proportional hazards models to estimate sex-specific hazard ratios (HRs) and 95% confidence intervals (CI), and women-to-men ratio of HRs (RHR) for the association between CVD (coronary heart diseases (CHD), stroke, and heart failure) and incident dementia (all-cause dementia, Alzheimer's Disease (AD), and vascular dementia (VD)). The moderator roles of socioeconomic (education, income), lifestyle (smoking, BMI, leisure activities, and physical activity), genetic factors (*APOE* allele status), and medical history were also analyzed.

**Results:**

Compared to people who did not experience a CVD event, the HRs (95%CI) between CVD and all-cause dementia were higher in women compared to men, with an RHR (Female/Male) of 1.20 (1.13, 1.28). Specifically, the HRs for AD were higher in women with CHD and heart failure compared to men, with an RHR (95%CI) of 1.63 (1.39, 1.91) and 1.32 (1.07, 1.62) respectively. The HRs for VD were higher in men with heart failure than women, with RHR (95%CI) of 0.73 (0.57, 0.93). An interaction effect was observed between socioeconomic, lifestyle, genetic factors, and medical history in the sex-specific association between CVD and dementia.

**Conclusion:**

Women with CVD were 1.5 times more likely to experience AD than men, while had 15% lower risk of having VD than men.

**Supplementary Information:**

The online version contains supplementary material available at 10.1186/s13293-022-00431-5.

## Highlights

- Whether the incidence and timing of dementia is affected by an interaction of sex with cardiovascular diseases (CVD) events is unclear.
- We evaluated the sex-specific difference in dementia risk associated with CVDs, including its unique interaction with social, behavioral, genetic, and medical factors.
- Women with coronary heart disease and heart failure were 1.6 and 1.3 times more likely to develop Alzheimer’s disease than men with the same condition.
- Men with heart failure had 1.4 times higher risk of having vascular dementia than women.
- Socioeconomic, lifestyle, genetic, and medical factors moderated the sex-specific association between CVD and dementia.

**Supplementary Information:**

The online version contains supplementary material available at 10.1186/s13293-022-00431-5.

## Introduction

There is a significant sex difference in prevalence of dementia, especially in Alzheimer’s disease (AD) [1]. About two-thirds of AD patients are women [2]. Women show faster cognitive decline after diagnosis of mild cognitive impairment (MCI) or dementia, suggesting that sex is a crucial variable in disease severity and consequent heterogeneity [3].

Among women aged 60 years or older, studies showed a higher prevalence of AD and MCI than men of the same age [4]. These differences are not fully explained by societal and lifestyle risk factors [5]. Other factors may contribute to sex differences, including differences in longevity, biological factors (reproduction, sex hormones), gender roles and opportunities (education and income, leisure activities post-retirement), and medical factors [6]. For example, early surgically induced menopause has been shown to be associated with higher risk of cognitive decline and dementia [7], although the findings with natural menopause were inconsistent [8, 9].

Increasingly, cardiovascular diseases (CVD), including coronary heart disease (CHD), stroke, and heart failure, have been shown to affect the risk of developing vascular dementia (VD) [10] and AD [11]. Studies have examined the sex differences in the association between major cardiovascular risk factors (e.g., hypertension and diabetes) in midlife and dementia [12, 13], while sex differences between CVD events (i.e., CHD, stroke and heart failure) and dementia remain unclear. Further, in the association between CVD and dementia, there might be sex-specific interactions with social (e.g., education, income, and leisure activities), lifestyle (smoking, body mass index (BMI), leisure activities, and physical activities), genetic factors (apolipoprotein E (APOE) allele status), and medical history (hypertension and diabetes status). Accordingly, we designed this study to assess the sex-specific difference in dementia risk associated with CVDs, including its unique interaction with social, behavioral, genetic, and medical factors.

## Methods

### Participants

The UK Biobank is a large population-based prospective cohort study that recruited over 502,000 participants aged 40–70 years from 2006 to 2010. Individuals were invited to attend one of the 22 centers for baseline assessment. Written informed consent was obtained for collection of questionnaire and biological data. All participants were linked to hospital data and national death registries from England, Scotland, and Wales [12] to determine the date of the first diagnosis of CVD and dementia after the baseline assessment. UK Biobank received ethical approval from the UK National Health Service’s National Research Ethics Service (ref 11/NW/0382). This research was conducted under UK Biobank application number 68369. A prospective analysis was adopted based on participants with no dementia at baseline, and if a participant had dementia during follow-up and also experienced CVD, his/her diagnosis of CVD had to be in advance of dementia. This study is reported as per the Strengthening the Reporting of Observational Studies in Epidemiology (STROBE) guidelines (Additional file 1).

### Exposure and outcome variables

The exposure variable was the occurrence of first non-fatal CVD event (a composite of either incident CHD or heart failure or stroke). Physician-diagnosed CVD was ascertained from hospital medical records. When ascertained from hospital records, CHD was defined by the International Classification of Diseases 10th Edition (ICD-10) codes I21–I25, or defined by ICD-9 codes 410–413. Incident stroke was defined by the ICD-10 codes I60–I61, and I63–I64, or ICD-9 codes 430–434. Heart failure was defined by the ICD-10: I50.

The study endpoint was incident fatal or non-fatal all-cause dementia, including dementia subtypes of AD and VD. The ICD-10 codes F00, F01, F02, F03, G30, G31·0, G31·8 and ICD-9 code 290·1 were used to identify participants with all-cause dementia if one or more of these codes were recorded as a primary or secondary diagnosis in the health records. Incident AD was defined by ICD-10 codes F00, G30 and ICD-9 code 290·1. Incident VD was defined by ICD-10 code F01. Outcome adjudication for incident dementia was conducted by the UK Biobank Outcome Adjudication team.

### Covariates

We included the following factors in the analyses as covariates according to evidence from previous studies [11, 12]: age at baseline, race/ethnicity, years of education, income level, smoking status, physical activity strength, number of leisure activities, BMI, hypertension status, type 2 diabetes status, and *APOE* allele status. Race/ethnicity was categorized as white and non-white. Years of education was categorized as ≤ 10, 11–12, and > 12 years. Income level was divided into four categories of level 1 (Less than £18,000), level 2 (£18,000 to £30,999), level 3 (£31,000 to £51,999), and level 4 (greater than 52,000). Smoking status was categorized as current, former, or never smokers. Physical activity level was categorized as light (< 600 metabolic equivalent (MET)-min/week), moderate (600 to < 3000 MET-min/week), and high (≥ 3000 MET-min/week) based on standard scoring criteria. Number of leisure activities was categorized as none, one, and two or more. BMI was categorized according to the World Health Organization criteria as < 25 kg/m^2^, 25 to 29.9 kg/m^2^, and ≥ 30 kg/m^2^. Hypertension or diabetes status was dichotomized as present or absent based on self-report at baseline. *APOE* allele status was based on two single nucleotide polymorphisms (SNPs): rs7412 and rs429358. Participants with *APOE* e4 allele (e3/e4, e4/e4, and occasionally e2/e4 genotypes) were compared with those with the e2/e2, e2/e3, or e3/e3 genotype.

### Statistical analyses

Baseline characteristics are presented as means and standard deviation (SD) for continuous variables and as percentages (%) for categorical variables. Cox proportional hazards regression models were used to estimate the sex-specific hazard ratios (HR) and 95% confidence intervals (CI) between CVD (including any CVD, CHD, stroke, and heart failure) and dementia (including all-cause dementia, AD, and VD). The proportional hazards (PH) assumption was tested graphically using a plot of the log cumulative hazard, where the logarithm of time is plotted against the estimated log cumulative hazard. The curves for the two CVD status (experienced or not) were approximately parallel; thus, the PH assumption was deemed reasonable. The interaction term between CVD types and sex was used to obtain the women-to-men ratio of hazard ratios (RHR) for each dementia type and the type of CVD event. Hospital inpatient data and death data were censored on the 30 January 2021 or when death, fatal, or non-fatal dementia was recorded. For participants who experienced a dementia, follow-up time was calculated as their age when dementia was diagnosed minus baseline age; for participants without experiencing dementia, follow-up time was defined as their age at last follow-up (censored date) minus baseline age. We first analyzed CVD types and incident all-cause dementia, followed by separate analyses for incident AD and VD. HRs (95% CI) were adjusted for age at baseline, race/ethnicity, years of education, income level, smoking status, physical activity level, number of leisure activities, BMI, hypertension status, type 2 diabetes status, and *APOE4* allele status.

### Subgroup analysis and sensitivity analysis

To examine whether timing of CVD occurrence moderates the association between CVD and dementia, we divided people with CVD into two categories of age of first CVD < 65 years and ≥ 65 years. Also, to examine the association between CVD and timing of dementia, people were separated by age of diagnosis of dementia < 75 and ≥ 75 years. In addition, we also examined the role of gender related social factors (i.e., education, income, and leisure activities), lifestyle factors (smoking, BMI, and physical activities), genetic factors (number of *APOE* e4 allele), and medical history (hypertension and diabetes status) on the sex-specific association between CVD and all-cause dementia by combining these factors with sex and CVD. These factors with each type of CVD and dementia subtypes were also analyzed. Finally, to avoid reverse causation, we performed a sensitivity analysis, including CVD events which occurred at least 3 and 5 years before dementia.

## Results

### Characteristics of participants (Table 1)

**Table 1:** Characteristics of participants by sex, CVD and dementia experienced or not, *n* (%)

Characteristics	N	CVD experienced or not				Dementia experienced or not			
		Female (*n* = 251,039)		Male (*n* = 213,577)		Female (*n* = 251 039)		Male (*n* = 213,577)	
		No (*n* = 239,138)	Yes (*n* = 11,901)	No (*n* = 189,827)	Yes (*n* = 23,750)	No (*n* = 248,461)	Yes (*n* = 2578)	No (*n* = 210,628)	Yes (*n* = 2949)
Race/ethnicity									
White	440,922	227,070 (51.5)	11,267 (2.6)	179,911 (40.8)	22,674 (5.1)	235,855 (53.5)	2482 (0.6)	199,760 (45.3)	2825 (0.6)
Non-White	23,694	12,068 (50.9)	634 (2.7)	9916 (41.9)	1076 (4.5)	12,606 (53.2)	96 (0.4)	10,868 (45.9)	124 (0.5)
Education level (years)									
< = 10	228,838	115,524 (50.5)	7674 (3.4)	91,069 (39.8)	14,571 (6.4)	121,590 (53.1)	1608 (0.7)	103,844 (45.4)	1796 (0.8)
11–12	55,721	30,873 (55.4)	1170 (2.1)	21,461 (38.5)	2217 (4)	31,763 (57.0)	280 (0.5)	23,394 (42)	284 (0.5)
> 12	180,057	92,741 (51.5)	3057 (1.7)	77,297 (42.9)	6962 (3.9)	95,108 (52.8)	690 (0.4)	83,390 (46.3)	869 (0.5)
Physical activity level (MET)									
Light (< 600)	105,714	55,336 (52.4)	3395 (3.2)	40,892 (38.7)	6091 (5.8)	58,142 (55)	589 (0.6)	46,295 (43.8)	688 (0.7)
Moderate (600–3000)	186,389	99,987 (53.6)	4710 (2.5)	72,402 (38.8)	9290 (5)	103,610 (55.6)	1087 (0.6)	80,512 (43.2)	1180 (0.6)
High (≥ 3000)	172,513	83,815 (48.6)	3796 (2.2)	76,533 (44.4)	8369 (4.9)	86,709 (50.3)	902 (0.5)	83,821 (48.6)	1081 (0.6)
Income level (£)									
Less than 18,000	104,445	57,177 (54.7)	4994 (4.8)	34,189 (32.7)	8085 (7.7)	61,014 (58.4)	1157 (1.1)	41,134 (39.4)	1140 (1.1)
18,000 to 30,999	113,933	60,359 (53)	3156 (2.8)	43,862 (38.5)	6556 (5.8)	62,825 (55.1)	690 (0.6)	49,542 (43.5)	876 (0.8)
31,000 to 51,999	120,669	61,580 (51)	2044 (1.7)	52,124 (43.2)	4921 (4.1)	63,212 (52.4)	412 (0.3)	56,514 (46.8)	531 (0.4)
Greater than 52,000	125,569	60,022 (47.8)	1707 (1.4)	59,652 (47.5)	4188 (3.3)	61,410 (48.9)	319 (0.3)	63,438 (50.5)	402 (0.3)
No. of leisure activities									
No	131,434	67,325 (51.2)	3877 (3)	52,972 (40.3)	7260 (5.5)	70,384 (53.6)	818 (0.6)	59,303 (45.1)	929 (0.7)
One	202,587	101,469 (50.1)	4987 (2.5)	85,320 (42.1)	10,811 (5.3)	105,303 (52)	1153 (0.6)	94,739 (46.8)	1392 (0.7)
Two or more	130,595	70,344 (53.9)	3037 (2.3)	51,535 (39.5)	5679 (4.4)	72,774 (55.7)	607 (0.5)	56,586 (43.3)	628 (0.5)
Body mass index (kg/m^2^)									
Underweight < 18.5	2398	1847 (77.0)	68 (2.8)	445 (18.6)	38 (1.6)	1887 (78.7)	28 (1.2)	470 (19.6)	13 (0.5)
Normal [18.5,25.0)	153,058	96,724 (63.2)	2739 (1.8)	49,653 (32.4)	3942 (2.6)	98,551 (64.4)	912 (0.6)	52,847 (34.5)	748 (0.5)
Overweight [25.0,30.0)	198,230	87,839 (44.3)	4369 (2.2)	95,114 (48)	10,908 (5.5)	91,227 (46)	981 (0.5)	104,683 (52.8)	1339 (0.7)
Obese > = 30	110,930	52,728 (47.5)	4725 (4.3)	44,615 (40.2)	8862 (8)	56,796 (51.2)	657 (0.6)	52,628 (47.4)	849 (0.8)
Smoking status									
Never	254,650	143,769 (56.5)	5821 (2.3)	96,819 (38)	8241 (3.2)	148,187 (58.2)	1403 (0.6)	103,917 (40.8)	1143 (0.5)
Past	162,083	74,956 (46.3)	4582 (2.8)	70,301 (43.4)	12,244 (7.6)	78,601 (48.5)	937 (0.6)	81,077 (50)	1468 (0.9)
Current	47,883	20,413 (42.6)	1498 (3.1)	22,707 (47.4)	3265 (6.8)	21,673 (45.3)	238 (0.5)	25,634 (53.5)	338 (0.7)
Diabetes status									
No	440,477	231,924 (52.7)	10,368 (2.4)	178,735 (40.6)	19,450 (4.4)	239,963 (54.5)	2329 (0.5)	195,749 (44.4)	2436 (0.6)
Yes	24,139	7214 (29.9)	1533 (6.4)	11,092 (46)	4300 (17.8)	8498 (35.2)	249 (1)	14,879 (61.6)	513 (2.1)
Hypertension status									
No	336,017	183,668 (54.7)	4749 (1.4)	136,517 (40.6)	11,083 (3.3)	186,945 (55.6)	1472 (0.4)	145,983 (43.5)	1617 (0.5)
Yes	128,599	55,470 (43.1)	7152 (5.6)	53,310 (41.5)	12,667 (9.9)	61,516 (47.8)	1106 (0.9)	64,645 (50.3)	1332 (1)
APOE									
No apoE4	112,993	56,186 (49.7)	3135 (2.8)	47,608 (42.1)	6064 (5.4)	58,874 (52.1)	447 (0.4)	53,030 (46.9)	642 (0.6)
One apoE4	342,293	178,142 (52)	8535 (2.5)	138,430 (40.4)	17,186 (5)	184,815 (54)	1862 (0.5)	153,568 (44.9)	2048 (0.6)
Two apoE4	9330	4810 (51.6)	231 (2.5)	3789 (40.6)	500 (5.4)	4772 (51.2)	269 (2.9)	4030 (43.2)	259 (2.8)

Overall, 464,616 participants were included in the analysis, with a mean (SD) age at baseline of 56.6 (8.1) years, and 54.0% of them were females. A majority of them (94.9%) were White individuals. The mean (SD) follow-up was 11.2 (1.5) years. The crude incidence rates of all-cause dementia were 9.2 for women and 12.4 for men, per 10,000 person-years. In both males and females, the incidence of dementia was lower in people with higher educational level, higher income level, and a greater number of leisure activities, while the incidence was higher in people with diabetes, hypertension, and two apoE4 alleles. Besides, in females, the prevalence of dementia was higher in underweight women than in normal or overweight/obese women. In males, the prevalence of dementia was higher in ever smokers.

### CVD events and dementia (Table 2)

**Table 2:** Sex-specific hazard ratios (HRs) and 95%CIs between cardiovascular disease (CVD) events and dementia subtypes: a prospective analysis

Dementia	Sex	Did not experience any CVD event	Experienced any CVD	Only experienced CHD	Only experienced stroke	Only experienced heart failure
All-cause dementia	All participants	Reference	2.20 (2.06, 2.35)	1.69 (1.53, 1.86)	2.37 (2.05, 2.75)	2.19 (1.95, 2.46)
	Female	Reference	2.31 (2.07, 2.57)	1.84 (1.55, 2.18)	2.40 (1.91, 3.02)	2.10 (1.73, 2.54)
	Male	Reference	2.01 (1.85, 2.18)	1.52 (1.35, 1.71)	2.24 (1.86, 2.71)	2.14 (1.85, 2.47)
	Ratio of HR (female/male)		1.20 (1.13, 1.28)	1.28 (1.15, 1.42)	1.12 (0.97, 1.31)	1.00 (0.89, 1.13)
Alzheimer's disease	All participants	Reference	1.71 (1.53, 1.90)	1.50 (1.28, 1.75)	1.48 (1.12, 1.95)	1.57 (1.28, 1.92)
	Female	Reference	2.07 (1.75, 2.45)	1.92 (1.50, 2.46)	1.44 (0.93, 2.23)	1.76 (1.30, 2.40)
	Male	Reference	1.46 (1.26, 1.68)	1.25 (1.03, 1.53)	1.44 (1.00, 2.06)	1.39 (1.05, 1.82)
	Ratio of HR (female/male)		1.50 (1.34, 1.67)	1.63 (1.39, 1.91)	1.06 (0.80, 1.41)	1.32 (1.07, 1.62)
Vascular dementia	All participants	Reference	3.37 (2.98, 3.82)	2.01 (1.66, 2.45)	5.01 (4.00, 6.27)	2.99 (2.41, 3.72)
	Female	Reference	2.77 (2.21, 3.47)	1.94 (1.34, 2.79)	4.13 (2.78, 6.14)	2.27 (1.52, 3.41)
	Male	Reference	3.35 (2.87, 3.91)	1.89 (1.50, 2.38)	5.24 (3.98, 6.91)	3.22 (2.49, 4.16)
	Ratio of HR (Female/Male)		0.86 (0.75, 0.98)	1.05 (0.84, 1.30)	0.82 (0.64, 1.04)	0.73 (0.57, 0.93)

All HRs were adjusted for age at baseline, race/ethnicity, educational years, income level, physical activity level, leisure activities, body mass index (BMI), smoking status, diabetes status, hypertension status, and APOE

Compared to people who did not experience any CVD event, people who experienced CVD had higher risk of all-cause dementia, AD, and VD, with HRs (95% CI) of 2.20 (2.06, 2.35), 1.71 (1.53, 1.90), and 3.37 (2.98, 3.82) respectively. The association of CVD with AD was higher in women (HR 2.07, 95% CI: 1.75–2.45) than in men (1.46, 1.26–1.68), with an RHR (Female/Male) of 1.50 (1.34, 1.67). After the relationships between specific types of CVD events and AD were analyzed, the sex difference was only observed for the association between CHD and AD, or heart failure and AD, with RHR (Female/Male) 95% CI of 1.63 (1.39, 1.91) and 1.32 (1.07, 1.62), respectively. In contrast to the sex difference between CVD and AD, the association of overall CVD with VD was higher in men (3.35, 2.87–3.91) than in women (2.77, 2.21–3.47), with an RHR (female/male) of 0.86 (0.75, 0.98). After types of CVD and VD were analyzed, the sex difference was only observed between heart failure and VD, with an RHR (95% CI) (female/male) of 0.73 (0.57, 0.93). Sensitivity analysis, including CVD events which occurred at least three years or five before occurrence of dementia, showed consistent results (Additional file 1: Tables S1 and S2).

### Subgroup analyses by age when experienced CVD, socioeconomic, lifestyle, genetic, and medical factors

**Fig. 1:**
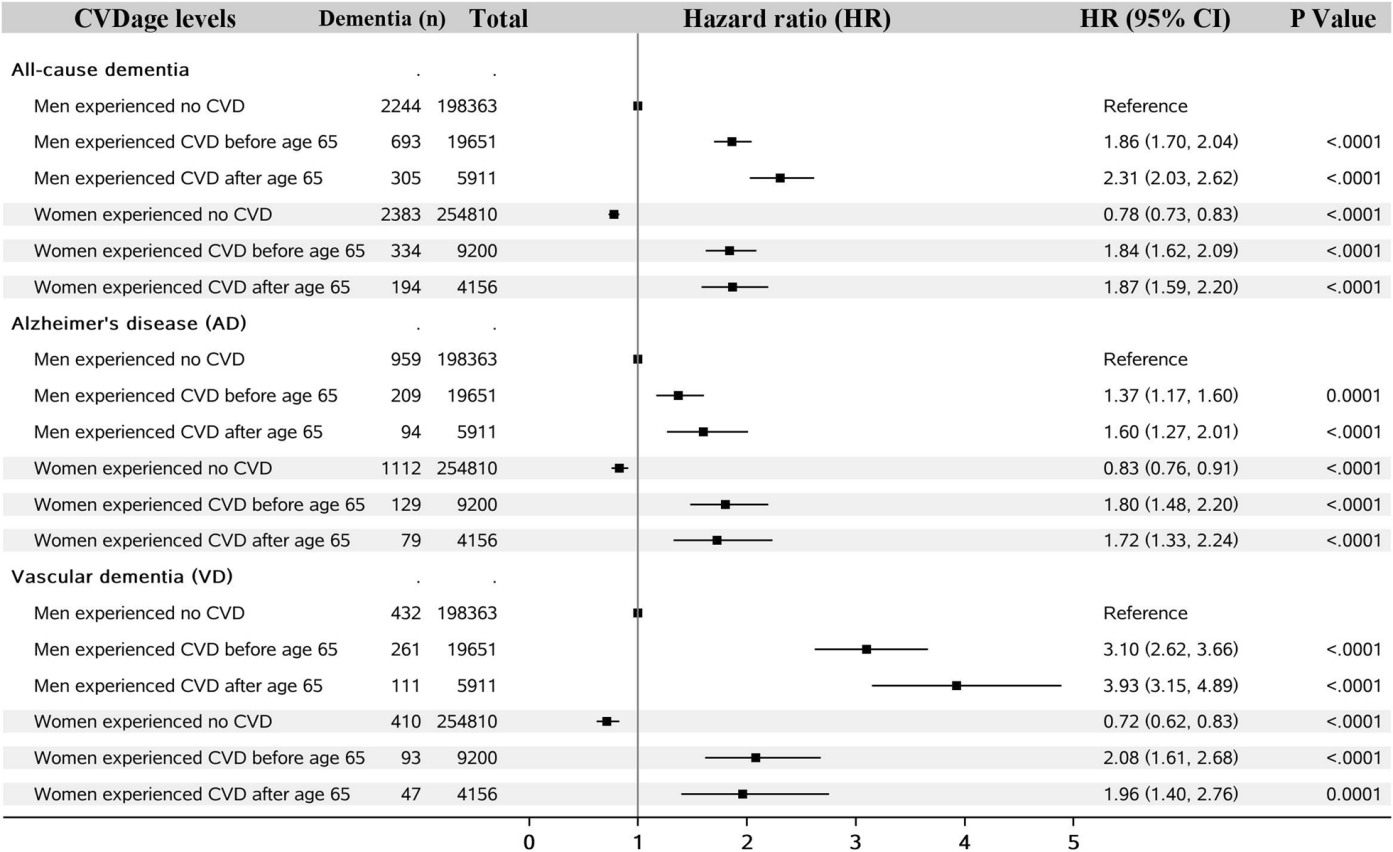
Sex differences in the association between cardiovascular diseases (CVD) and dementia by age when experienced CVD

**Fig. 2:**
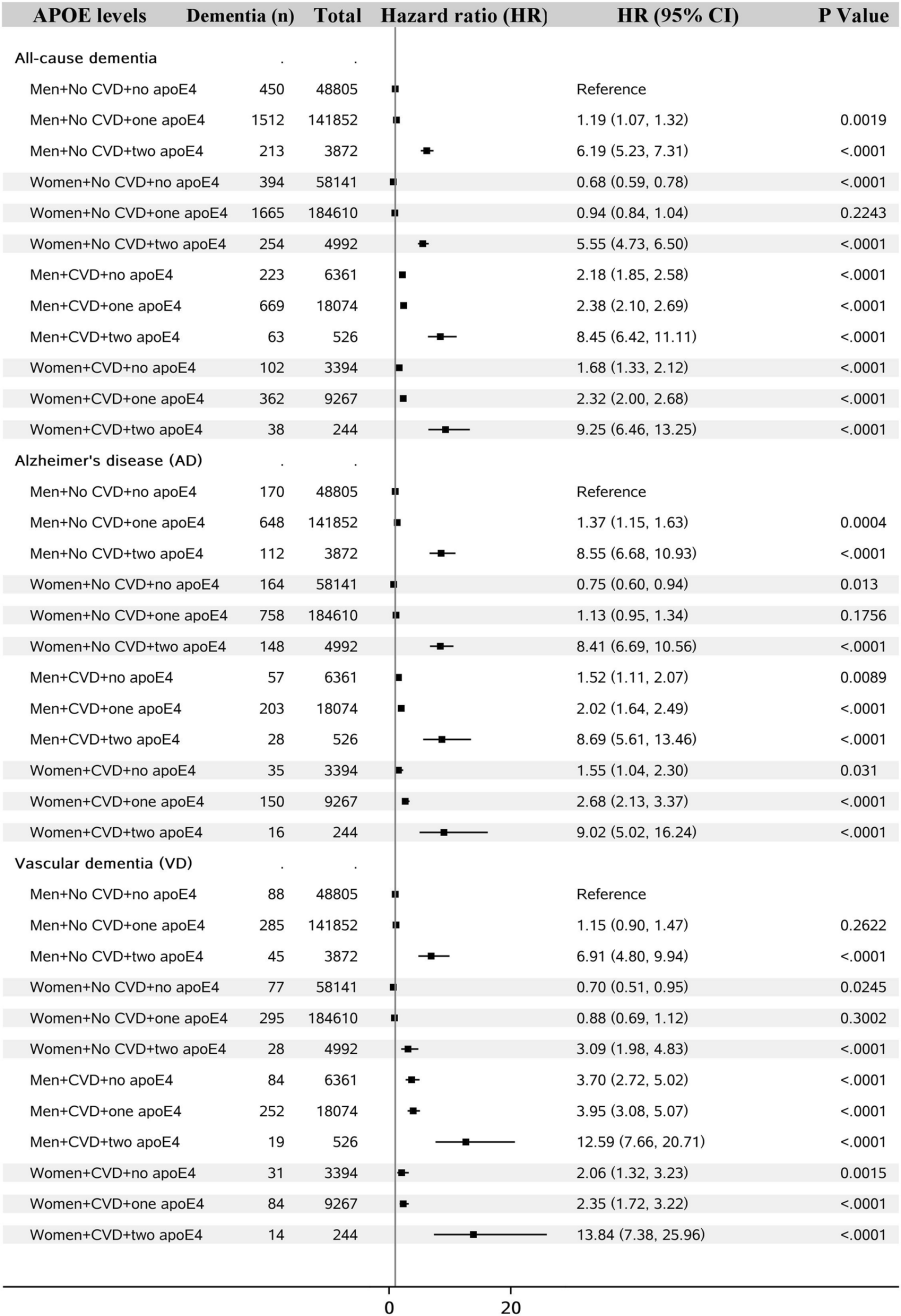
Sex differences in the association between cardiovascular diseases (CVD) and dementia by apoE4 status

**Fig. 3:**
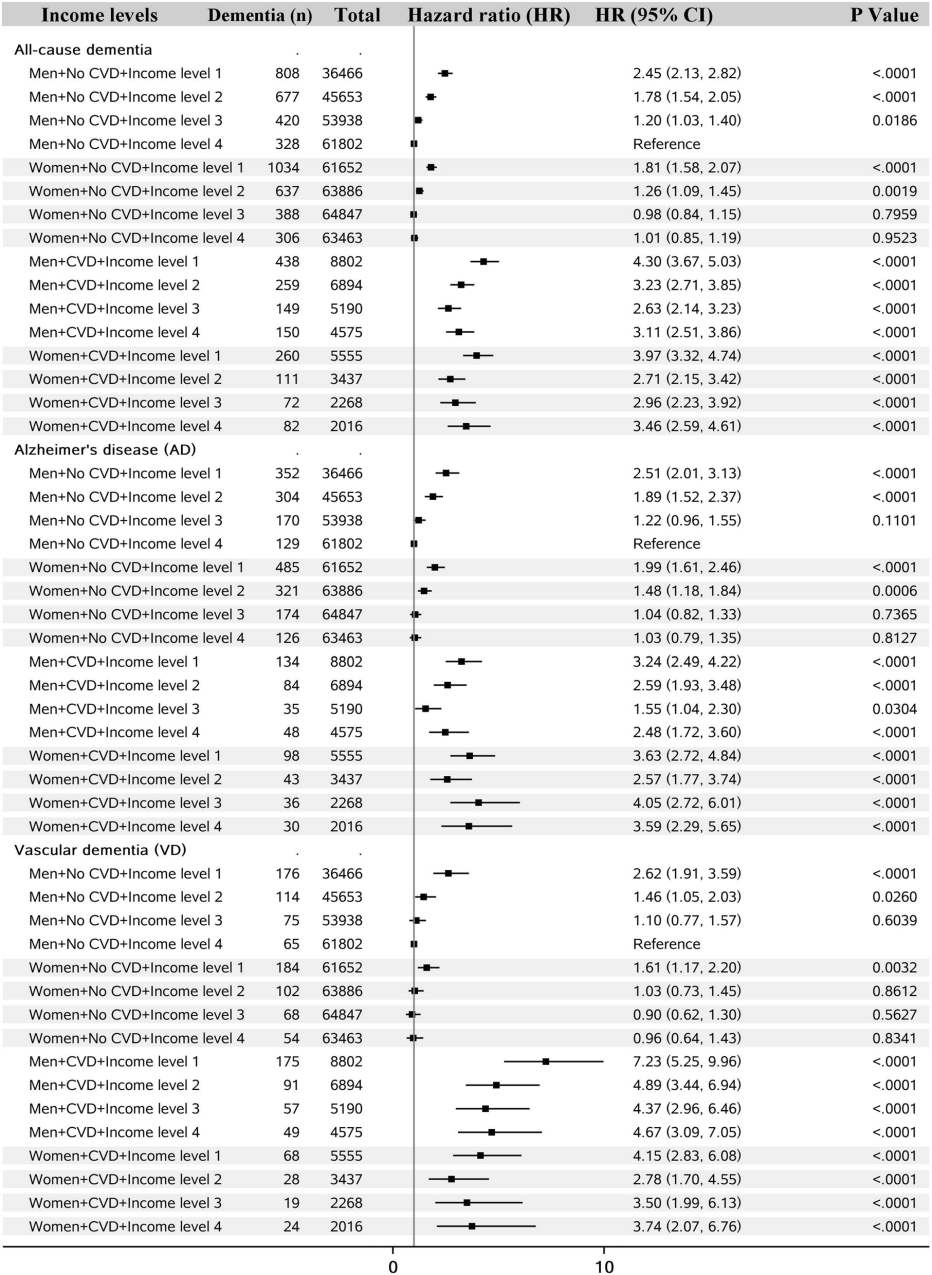
Sex differences in the association between cardiovascular diseases (CVD) and dementia by income levels

Analyses stratified by age when experienced CVD (before or after 65 years) showed that in women, the associations of CVD with AD and VD were not different by age at the onset of CVD, while men with CVD after age 65 had higher risk of AD and VD than those with CVD before age 65 (Fig. 1). The risk of dementia was much higher in people with two apoE4 alleles in both men and women. People who experienced CVD and had two apoE4 alleles had around 10 times higher risk of having dementia (Fig. 2). In people without CVD, higher income level was related to lower risk of dementia, while in people with CVD, a J-shape relationship was observed between income level and dementia (Fig. 3). Both lower and higher income levels were related to higher risk of dementia, but the increased risk was greater in those with lower incomes. The roles of education, leisure activity, BMI, smoking, physical activity, hypertension and diabetes status in CVDs, and dementia subtypes are shown in Additional file 1: Figs. S1–S7. In people with CVD, a J-shape relationship was observed between BMI level and VD. Both lower and higher BMI levels were related to higher risk of VD, but the increased risk was greater in those with lower BMI. When BMI was assessed in 10 years before dementia diagnosis (i.e., late-life BMI), greater BMI was related to lower risk of dementia (Additional file 1: Table S3). Higher number of leisure activities was consistently linked to lower risk of dementia, and no protective effect of education on VD was observed in CVD patients.

## Discussion

### CHD and dementia

A few systematic reviews and meta-analysis have examined the association between CHD and all-cause dementia and dementia subtypes [14, 15]. A recent review found that CHD was associated with 27% higher risk of all-cause dementia, and analyses with dementia subtypes showed that the significant association was only observed between CHD and VD (RR 1.34, 95% CI 1.28–1.39), but not with AD (0.99, 0.92–1.07) [14]. Similarly, Wolters FJ et al. reported that the association of CHD with all-cause dementia and AD were (1.27, 1.08–1.50) and (1.07, 0.90–1.28), respectively [15]. However, these reviews did not separate the association by sex and examine the sex difference. Consistent with prior studies, we found that CHD was linked to 65% higher risk of all-cause dementia, and significant associations were observed with dementia subtypes of both AD and VD. In addition, there was also sex differences in the relationship between CHD and all-cause dementia and AD. Women who experienced CHD was 1.60 times more likely to develop AD than men.

### Heart failure and dementia

A recent review found that the RR (95% CI) between heart failure and all-cause dementia, AD, was (1.59, 1.19–2.13) and (1.44, 0.95–2.16), respectively [15]. Similarly, Cannon et al. also reported that the risk for all-cause dementia and cognitive impairment in the heart failure population was 2.64 (95% 1.83–3.80) [16]. In line with the findings of previous studies, we observed a higher risk (HR 2.63, 95% CI 2.35–2.95) of all-cause dementia in people with heart failure. In contrast to previous reviews which reported no association between heart failure and AD (possibly due to the high heterogeneity among studies) [15, 17], we also found significant associations with AD (1.92, 1.56–2.35) and VD (3.67, 2.96–4.55). In addition, a clear sex difference was observed in the association between heart failure and VD. Compared to women with heart failure, men with heart failure were about 1.5 times (i.e., inverse of 0.68) more likely to develop VD.

### Stroke and dementia

A review by Kuzma et al. found that patients with prevalent stroke were 2.18 (1.90–2.50) times more likely to experience all-cause dementia [18]. Likewise, Zhu et al. reported a 2.40 times higher risk of all-cause dementia in people with stroke [19]. Consistent to previous findings, we observed that people who experienced stroke had 2.39 times higher risk of developing all-cause dementia. Further analyses showed that the elevated risk was mainly reflected in the association with VD (HR: 5.05 with VD vs 1.51 with AD). Evidence has shown that stroke survivors are at increased risk of developing post-stroke VD [20]. No substantial gender difference for the risk of AD and VD after stroke was found, in line with previous report [21].

### Mechanisms

Several mechanisms might contribute to the sex difference in the association between CVD and dementia. First, the development, manifestation, and complications of CVD may differ by sex [22], affecting the “heart-to-brain” connection. Heart diseases (e.g., heart failure) affect the cardiac output, leading to cerebral hypoperfusion [23]. The latter contributes to the formation of tau-containing neurofibrillary tangles and amyloid β (Aβ) plaques which characterize AD [24]. Research has shown heart failure with reduced ejection fraction (EF) more frequently affects men, and heart failure with preserved EF more frequently affects women [25]. Reduced EF have exaggerated reductions in cerebral blood flow [26]. This may explain why men with heart failure had greater risk of VD than women in our finding. In addition, myocardial infarctions (MI) are more severe in women than in men. In the first year after MI, women are 1.5 times more likely to die compared to their male counterparts [27]. Further, women were more likely than men to be older and have a more complicated medical history at the time of their MI [22], which may also affect the heart-to-brain connection in women. Second, there might be an interaction between sex, cardiovascular, and genetic risk factors, which are all related to risk of CVD and dementia. Hypertension in midlife increased the risk of dementia among women only, although hypertension was more prevalent among men in midlife [13]. Also, depression and sleep disorders, both risk factors for AD, are also known to be more prevalent in women [28]. Sex also modulates the susceptibility to AD conferred by APOE genotype. APOE e4 was associated with a higher risk of AD in females than in males [29]. Also, there was a strong association between APOE 4 (ε3ε4 and ε4ε4) and CHD [30], indicating APOE 4 might be a confounder in the relationship between CHD and AD. Thus, the sex difference association between CVD and AD might be cofounder by APOE4. Nevertheless, the APOE4 allele status was adjusted in our analyses. The sex difference between the two cannot be fully explained by the sex differences in the association between APOE4 allele and AD. Last, some sex-specific risk factors might play a role in the observed sex differences. Pre-eclampsia has been associated with higher risks of cardiovascular disease, cognitive impairment later in life, and protein misfolding with defective amyloid processing [3]. Early menopause (either natural or surgical menopause) has been associated with higher risks of cognitive decline, and dementia and 1.5–2 times elevated risk of CHD and stroke [31, 32]. These female-specific factors confer excess risk to both cardiovascular diseases and AD in women.

### Socioeconomic, lifestyle, and medical risk factors

Higher education and income, more leisure activities, and greater physical activity are viewed as protective factors for both CVD and dementia, and women in older cohorts often had less educational attainment and physical activity opportunity [3]. We found in people with no CVD, higher education was protective against dementia, while no clear protective effect was observed in people with CVD (especially for VD). In people without experiencing CVD, we found higher income was associated with lower risk of dementia, while in people who experienced CVD, there was a J-shape relationship between income level and dementia. It is possible that people with higher income and heart disease may have more opportunity to be diagnosed earlier if they had dementia [33].

Either with or without CVD, we observed a consistent trend that more leisure activities had lower risk of dementia. Besides, the protective effect of leisure activity is more evident in male CVD patients than females in the association with VD. Although some studies found physical activity at midlife is associated with a decreased risk of AD [6], we did not observe a clear association between them. The evidence between the BMI and dementia is still mixed. Two million-size population studies found that risk of dementia decreased with the growing BMI category, and per 5-kg/m^2^ increase in BMI was linked to 29% lower risk of dementia [34, 35]. We also found greater BMI was associated with less risk of AD in both men and women, not interacted by CVD experienced or not. Current smokers had elevated risk of dementia, especially for VD in men who experienced CVD. Consistent with Gong J et al. study [12], no clear interplay of diabetes on the relationship between CVD and all-cause dementia was observed, though risk of VD was higher in men than women with CVD and diabetes.

### Strengths and limitations

One strength of our study was the large sample size which enable us to explore the relationship between CVD subtypes and dementia subtypes, considering joint effect with socio-behavioral and biological factors. Also, both CVD events and dementia outcomes were ascertained through linkage to medical or insurance records, avoiding self-reported bias. To the best of our knowledge, this is the first study to systematically evaluate the sex difference in association between CVD and dementia subtypes, and examine the roles of socioeconomic, lifestyle, genetic, and medical factors in their associations. Our study also has several limitations. When compared the sex-specific association of CVD and dementia, we did not consider the severity of a certain CVD event in men and women. MI is generally more severe in women than in men over 65 years of age [36]. The association of CHD with dementia in females might be underestimated. Also, there might be some other confounding factors that co-drive the relationship between CVD and dementia, e.g., diet. In addition, the observed relationship is limited to people of Caucasian ancestry, which may limit the extrapolation of the results.

### Perspectives and significance

Women with CVD had higher risk of developing all-cause dementia than men. The risk for AD was greater in women with CHD and heart failure than in men, while the risk for VD was greater in men with heart failure than in women. There is no current cure for dementia, identifying sex-specific at-risk populations after experiencing CVDs is essential for adopting sex-sensitive strategies for secondary prevention of dementia. Further research to explore the sex differences in CVD, including the differences in clinical symptoms and treatment, may help understand the sex-specific association between CVD and dementia.

## Supplementary Information

**Additional file 1.** Additional information about the results of CVD events which occurred at least three years or five before occurrence of dementia and the roles of education, leisure activity, BMI, smoking, physical activity, hypertension, and diabetes status in CVDs and dementia subtypes. **Table S1.** Sex-specific hazard ratios (HRs) between cardiovascular disease and dementia subtypes (CVD events occurred at least three years before dementia): sensitivity analysis. **Table S2.** Sex-specific hazard ratios (HRs) between cardiovascular disease and dementia subtypes (CVD events occurred at least five years before dementia): sensitivity analysis. **Figure S1.** Sex differences in the association between cardiovascular diseases (CVD) and dementia by educational years. **Figure S2.** Sex differences in the association between cardiovascular diseases (CVD) and dementia by number of leisure activities. **Figure S3.** Sex differences in the association between cardiovascular diseases (CVD) and dementia by body mass index (BMI). **Figure S4.** Sex differences in the association between cardiovascular diseases (CVD) and dementia by smoking status. **Figure S5.** Sex differences in the association between cardiovascular diseases (CVD) and dementia by physical activities. **Figure S6.** Sex differences in the association between cardiovascular diseases (CVD) and dementia by diabetes status. **Figure S7.** Sex differences in the association between cardiovascular diseases (CVD) and dementia by hypertension status.

## Data Availability

UK Biobank data are available via www.ukbiobank.ac.uk. Syntax for the generation of derived variables and for the analysis used for this study will be submitted to UK Biobank for record.
