# Supplementary material for: Sex differences in the association between cardiovascular diseases and dementia subtypes: a prospective analysis of 464,616 UK Biobank participants
Source: Biol Sex Differ. 2022 May 7;13:21. doi: 10.1186/s13293-022-00431-5 (PMC9080133; doi:10.1186/s13293-022-00431-5)
Supplement: Supplementary file 1 — Additional file 1. Additional information about the results of CVD events which occurred at least three years or five before occurrence of dementia and the roles of education, leisure activity, BMI, smoking, physical activity, hypertension, and diabetes status in CVDs and dementia subtypes. Table S1. Sex-specific hazard ratios (HRs) between cardiovascular disease and dementia subtypes (CVD events occurred at least three years before dementia): sensitivity analysis. Table S2. Sex-specific hazard ratios (HRs) between cardiovascular disease and dementia subtypes (CVD events occurred at least five years before dementia): sensitivity analysis. Figure S1. Sex differences in the association between cardiovascular diseases (CVD) and dementia by educational years. Figure S2. Sex differences in the association between cardiovascular diseases (CVD) and dementia by number of leisure activities. Figure S3. Sex differences in the association between cardiovascular diseases (CVD) and dementia by body mass index (BMI). Figure S4. Sex differences in the association between cardiovascular diseases (CVD) and dementia by smoking status. Figure S5. Sex differences in the association between cardiovascular diseases (CVD) and dementia by physical activities. Figure S6. Sex differences in the association between cardiovascular diseases (CVD) and dementia by diabetes status. Figure S7. Sex differences in the association between cardiovascular diseases (CVD) and dementia by hypertension status. [file 13293_2022_431_MOESM1_ESM.docx]

**Additional file 1**

**Sex differences in the association between cardiovascular diseases and dementia subtypes: a prospective analysis of 464 616 UK Biobank participants**

**Authors**

Caiyun Dong, Chunmiao Zhou, Chunying Fu, Wenting Hao, Akihiko Ozaki, Nipun Shrestha, Salim S. Virani, Shiva Raj Mishra, Dongshan Zhu

**Correspondence**

Dongshan Zhu,

Department of Epidemiology, School of Public Health, Cheeloo College of Medicine, Shandong University, Jinan, China

44 Wenhuaxi Road, Jinan 250012, Shandong, China.

Email: [dongshan.zhu@uq.net.au](mailto:dongshan.zhu@uq.net.au)

**List**

**Table S1:** Sex-specific hazard ratios (HRs) between cardiovascular disease and dementia subtypes (CVD events occurred at least three years before dementia): sensitivity analysis

**Table S2:** Sex-specific hazard ratios (HRs) between cardiovascular disease and dementia subtypes (CVD events occurred at least five years before dementia): sensitivity analysis

**Table S3:** Sex differences in the association between body mass index (BMI) and dementia separated by obesity onset relative to dementia

**Table S4:** Characteristics of participants by sex in baseline

**Figure S1:** Sex differences in the association between cardiovascular diseases (CVD) and dementia by educational years.

**Figure S2:** Sex differences in the association between cardiovascular diseases (CVD) and dementia by number of leisure activities.

**Figure S3:** Sex differences in the association between cardiovascular diseases (CVD) and dementia by body mass index (BMI).

**Figure S4:** Sex differences in the association between cardiovascular diseases (CVD) and dementia by smoking status.

**Figure S5:** Sex differences in the association between cardiovascular diseases (CVD) and dementia by physical activities.

**Figure S6:** Sex differences in the association between cardiovascular diseases (CVD) and dementia by diabetes status.

**Figure S7:** Sex differences in the association between cardiovascular diseases (CVD) and dementia by hypertension status.

| **Additional Tables**  **TABLE S1.** Sex-specific hazard ratios (HRs) between cardiovascular disease and dementia subtypes (CVD events occurred at least three years before dementia): sensitivity analysis* | | | | | | |
| --- | --- | --- | --- | --- | --- | --- |
| Dementia | Sex | Did not experience any CVD event | Experienced any CVD | Only experienced CHD | Only experienced stroke | Only experienced heart failure |
| All-cause dementia | All participants | Reference | 1.83 (1.71, 1.96) | 1.68 (1.52, 1.85) | 2.37 (2.05, 2.75) | 0.73 (0.60, 0.88) |
|  | Female | Reference | 1.86 (1.65, 2.09) | 1.80 (1.52, 2.14) | 2.40 (1.91, 3.02) | 0.62 (0.44, 0.87) |
|  | Male | Reference | 1.70 (1.56, 1.86) | 1.52 (1.35, 1.71) | 2.24 (1.86, 2.71) | 0.75 (0.59, 0.95) |
|  | Ratio of HR (Female/Male) |  | 1.15 (1.07, 1.24) | 1.25 (1.13, 1.39) | 1.12 (0.97, 1.31) | 0.85 (0.69, 1.05) |
| Alzheimer's disease | All participants | Reference | 1.44 (1.28, 1.62) | 1.50 (1.28, 1.75) | 1.48 (1.12, 1.95) | 0.46 (0.32, 0.67) |
|  | Female | Reference | 1.69 (1.41, 2.02) | 1.93 (1.50, 2.47) | 1.44 (0.93, 2.23) | 0.46 (0.26, 0.84) |
|  | Male | Reference | 1.26 (1.08, 1.46) | 1.25 (1.03, 1.53) | 1.44 (1.00, 2.06) | 0.44 (0.27, 0.71) |
|  | Ratio of HR (Female/Male) |  | 1.43 (1.27, 1.61) | 1.63 (1.39, 1.91) | 1.06 (0.80, 1.41) | 1.11 (0.75, 1.63) |
| Vascular Dementia | All participants | Reference | 2.81 (2.46, 3.21) | 2.01 (1.66, 2.45) | 5.01 (4.00, 6.27) | 0.84 (0.57, 1.24) |
|  | Female | Reference | 2.31 (1.82, 2.94) | 1.94 (1.34, 2.79) | 4.13 (2.78, 6.14) | 0.71 (0.35, 1.43) |
|  | Male | Reference | 2.80 (2.38, 3.29) | 1.89 (1.50, 2.38) | 5.24 (3.98, 6.91) | 0.87 (0.55, 1.39) |
|  | Ratio of HR (Female/Male) |  | 0.86 (0.75, 0.99) | 1.05 (0.84, 1.30) | 0.82 (0.64, 1.04) | 0.84 (0.55, 1.29) |
| * All HRs were adjusted for age at last follow up, race/ethnicity, educational years, income level, physical activity level, leisure activities, body mass index (BMI), smoking status, diabetes status, hypertension and APOE. | | | | | | |

| **TABLE S2.** Sex-specific hazard ratios (HRs) between cardiovascular disease and dementia subtypes (CVD events occurred at least five years before dementia): sensitivity analysis * | | | | | | |
| --- | --- | --- | --- | --- | --- | --- |
| Dementia | Sex | Did not experience any CVD event | Experienced any CVD | Only experienced CHD | Only experienced stroke | Only experienced heart failure |
| All-cause dementia | All participants | Reference | 1.74 (1.62, 1.87) | 1.65 (1.50, 1.82) | 2.34 (2.02, 2.71) | 0.44 (0.34, 0.57) |
|  | Female | Reference | 1.74 (1.55, 1.97) | 1.74 (1.46, 2.07) | 2.37 (1.88, 2.98) | 0.34 (0.21, 0.54) |
|  | Male | Reference | 1.63 (1.49, 1.78) | 1.51 (1.34, 1.70) | 2.20 (1.82, 2.66) | 0.48 (0.36, 0.64) |
|  | Ratio of HR (Female/Male) |  | 1.13 (1.05, 1.21) | 1.22 (1.10, 1.36) | 1.13 (0.97, 1.31) | 0.73 (0.55, 0.97) |
| Alzheimer's disease | All participants | Reference | 1.37 (1.22, 1.54) | 1.46 (1.25, 1.71) | 1.42 (1.07, 1.88) | 0.28 (0.17, 0.45) |
|  | Female | Reference | 1.59 (1.32, 1.92) | 1.84 (1.43, 2.37) | 1.44 (0.93, 2.23) | 0.25 (0.11, 0.57) |
|  | Male | Reference | 1.20 (1.03, 1.40) | 1.24 (1.02, 1.51) | 1.34 (0.93, 1.95) | 0.29 (0.16, 0.52) |
|  | Ratio of HR (Female/Male) |  | 1.40 (1.25, 1.58) | 1.57 (1.34, 1.85) | 1.13 (0.85, 1.52) | 0.93 (0.56, 1.55) |
| Vascular Dementia | All participants | Reference | 2.73 (2.39, 3.12) | 2.00 (1.64, 2.43) | 5.01 (4.00, 6.27) | 0.56 (0.35, 0.90) |
|  | Female | Reference | 2.24 (1.75, 2.85) | 1.94 (1.35, 2.79) | 4.13 (2.78, 6.14) | 0.45 (0.18, 1.08) |
|  | Male | Reference | 2.73 (2.32, 3.21) | 1.86 (1.48, 2.35) | 5.24 (3.98, 6.91) | 0.60 (0.34, 1.05) |
|  | Ratio of HR (Female/Male) |  | 0.85 (0.74, 0.98) | 1.06 (0.85, 1.32) | 0.82 (0.64, 1.04) | 0.77 (0.45, 1.31) |
| * All HRs were adjusted for age at last follow up, race/ethnicity, educational years, income level, physical activity level, leisure activities, body mass index (BMI), smoking status, diabetes status, hypertension and APOE. | | | | | | |

| **TABLE S3.** Sex differences in the association between body mass index (BMI) and dementia separated by obesity onset relative to dementia | | | | | | | |
| --- | --- | --- | --- | --- | --- | --- | --- |
| Subgroup | Dementia | Total of number | Hazard ratio | Low | High | HR (95% CI) | p-value |
| obesity onset relative to dementia onset (<=10 years) | |  |  |  |  |  |  |
| Men+BMI<25 | 579 | 12743 | 1.000 | 1.000 | 1.000 | Reference |  |
| Men+BMI 25-29.9 | 960 | 24968 | 0.719 | 0.644 | 0.802 | 0.71 (0.64, 0.80) | <.0001 |
| Men+BMI>=30 | 608 | 13996 | 0.698 | 0.615 | 0.792 | 0.69 (0.61, 0.79) | <.0001 |
| Women+BMI<25 | 654 | 21695 | 1.000 | 1.000 | 1.000 | Reference |  |
| Women+BMI 25-29.9 | 693 | 20223 | 0.927 | 0.827 | 1.039 | 0.92 (0.82, 1.03) | 0.1948 |
| Women+BMI>=30 | 493 | 13608 | 0.799 | 0.699 | 0.913 | 0.79 (0.69, 0.91) | 0.001 |
| obesity onset relative to dementia onset (>10 years) | |  |  |  |  |  |  |
| Men+BMI<25 | 259 | 43705 | 1.000 | 1.000 | 1.000 | Reference |  |
| Men+BMI 25-29.9 | 508 | 85691 | 0.928 | 0.790 | 1.090 | 0.92 (0.79, 1.09) | 0.3639 |
| Men+BMI>=30 | 328 | 42822 | 0.902 | 0.750 | 1.084 | 0.90 (0.75, 1.08) | 0.2719 |
| Women+BMI<25 | 370 | 84928 | 1.000 | 1.000 | 1.000 | Reference |  |
| Women+BMI 25-29.9 | 412 | 78229 | 1.008 | 0.868 | 1.170 | 1.00 (0.86, 1.17) | 0.919 |
| Women+BMI>=30 | 289 | 49483 | 0.814 | 0.681 | 0.973 | 0.81 (0.68, 0.97) | 0.0237 |
| Note | | | | | | | |

| **TABLE S4.** Characteristics of participants by sex in baseline | | | | | |
| --- | --- | --- | --- | --- | --- |
| Characteristics | Female | | Male | | N |
|  | N | % | N | % |  |
| Race/ethnicity | 238337 | 54.05 | 202585 | 45.95 | 440922 |
| White |  |  |  |  |  |
| Non-White | 12702 | 53.61 | 10992 | 46.39 | 23694 |
| Education level (years) | 123198 | 53.84 | 105640 | 46.16 | 228838 |
| <=10 |  |  |  |  |  |
| 11-12 | 32043 | 57.51 | 23678 | 42.49 | 55721 |
| >12 | 95798 | 53.2 | 84259 | 46.8 | 180057 |
| Physical activity level | 58731 | 55.56 | 46983 | 44.44 | 105714 |
| Light |  |  |  |  |  |
| Moderate | 104697 | 56.17 | 81692 | 43.83 | 186389 |
| High | 87611 | 50.79 | 84902 | 49.21 | 172513 |
| Income level (£) | 62171 | 59.53 | 42274 | 40.47 | 104445 |
| Less than 18,000 |  |  |  |  |  |
| 18,000 to 30,999 | 63515 | 55.75 | 50418 | 44.25 | 113933 |
| 31,000 to 51,999 | 63624 | 52.73 | 57045 | 47.27 | 120669 |
| Greater than 52,000 | 61729 | 49.16 | 63840 | 50.84 | 125569 |
| No. of leisure activities | 71202 | 54.17 | 60232 | 45.83 | 131434 |
| No |  |  |  |  |  |
| One | 106456 | 52.55 | 96131 | 47.45 | 202587 |
| Two or more | 73381 | 56.19 | 57214 | 43.81 | 130595 |
| Body mass index (kg/m^2^) | 1915 | 79.86 | 483 | 20.14 | 2398 |
| Underweight<18.5 |  |  |  |  |  |
| Normal [18.5,25.0) | 99463 | 64.98 | 53595 | 35.02 | 153058 |
| Overweight [25.0,30.0) | 92208 | 46.52 | 106022 | 53.48 | 198230 |
| Obese>=30 | 57453 | 51.79 | 53477 | 48.21 | 110930 |
| Smoking status | 149590 | 58.74 | 105060 | 41.26 | 254650 |
| Never |  |  |  |  |  |
| Past | 79538 | 49.07 | 82545 | 50.93 | 162083 |
| Current | 21911 | 45.76 | 25972 | 54.24 | 47883 |
| Diabetes status | 242292 | 55.01 | 198185 | 44.99 | 440477 |
| No |  |  |  |  |  |
| Yes | 8747 | 36.24 | 15392 | 63.76 | 24139 |
| Hypertension status | 188417 | 56.07 | 147600 | 43.93 | 336017 |
| No |  |  |  |  |  |
| Yes | 62622 | 48.7 | 65977 | 51.3 | 128599 |
| APOE | 59321 | 52.5 | 53672 | 47.5 | 112993 |
| no apoE4 |  |  |  |  |  |
| one apoE4 | 186677 | 54.54 | 155616 | 45.46 | 342293 |
| two apoE4 | 5041 | 54.03 | 4289 | 45.97 | 9330 |
| Note |  |  |  |  |  |

**Additional Figures**


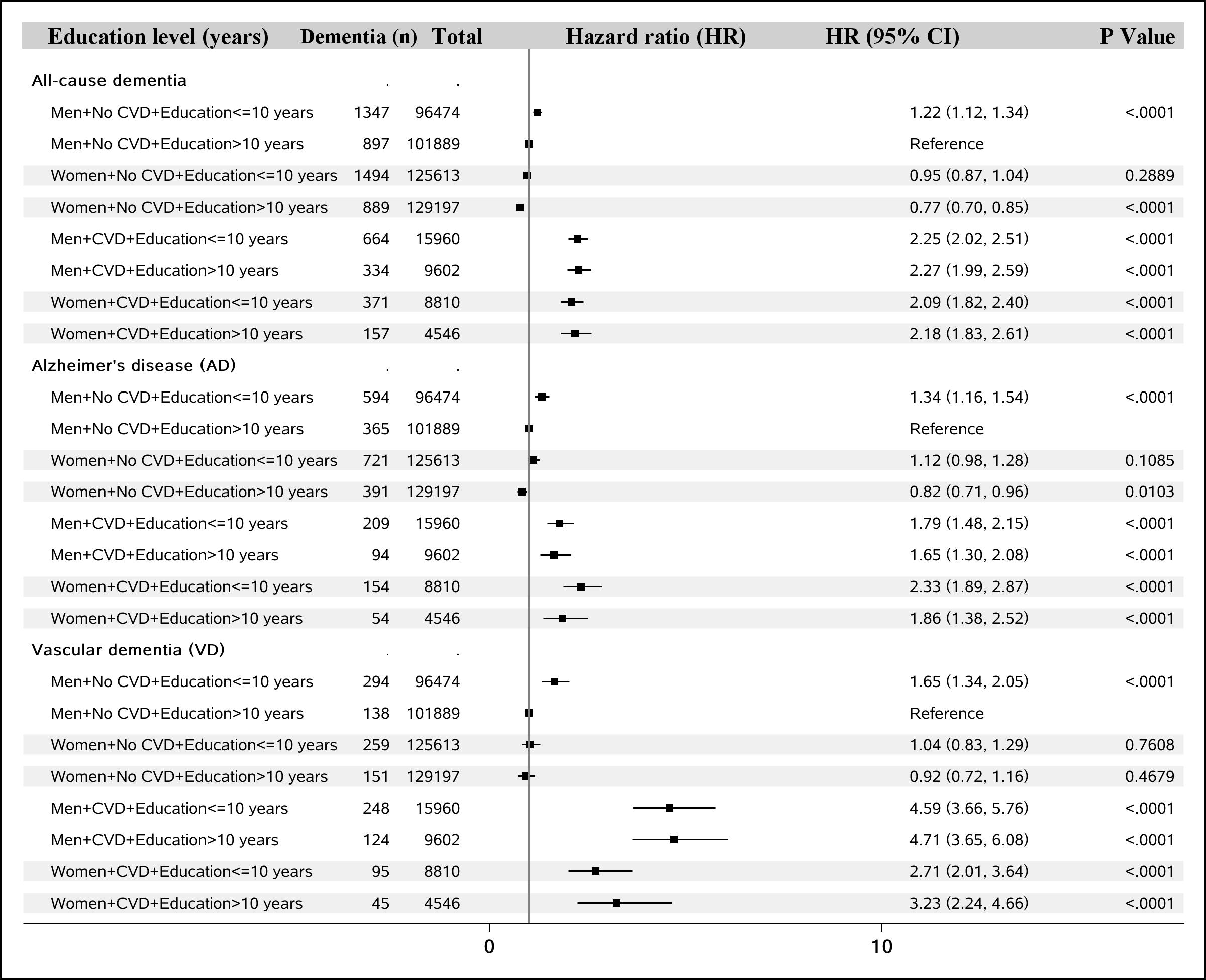


***FIGURE S1:* Sex differences in the association between cardiovascular diseases (CVD) and dementia by educational years.**

All HRs were adjusted for age at baseline, race/ethnicity, educational years, income level, physical activity level, leisure activities, body mass index (BMI), smoking status, diabetes status, hypertension status and APOE.


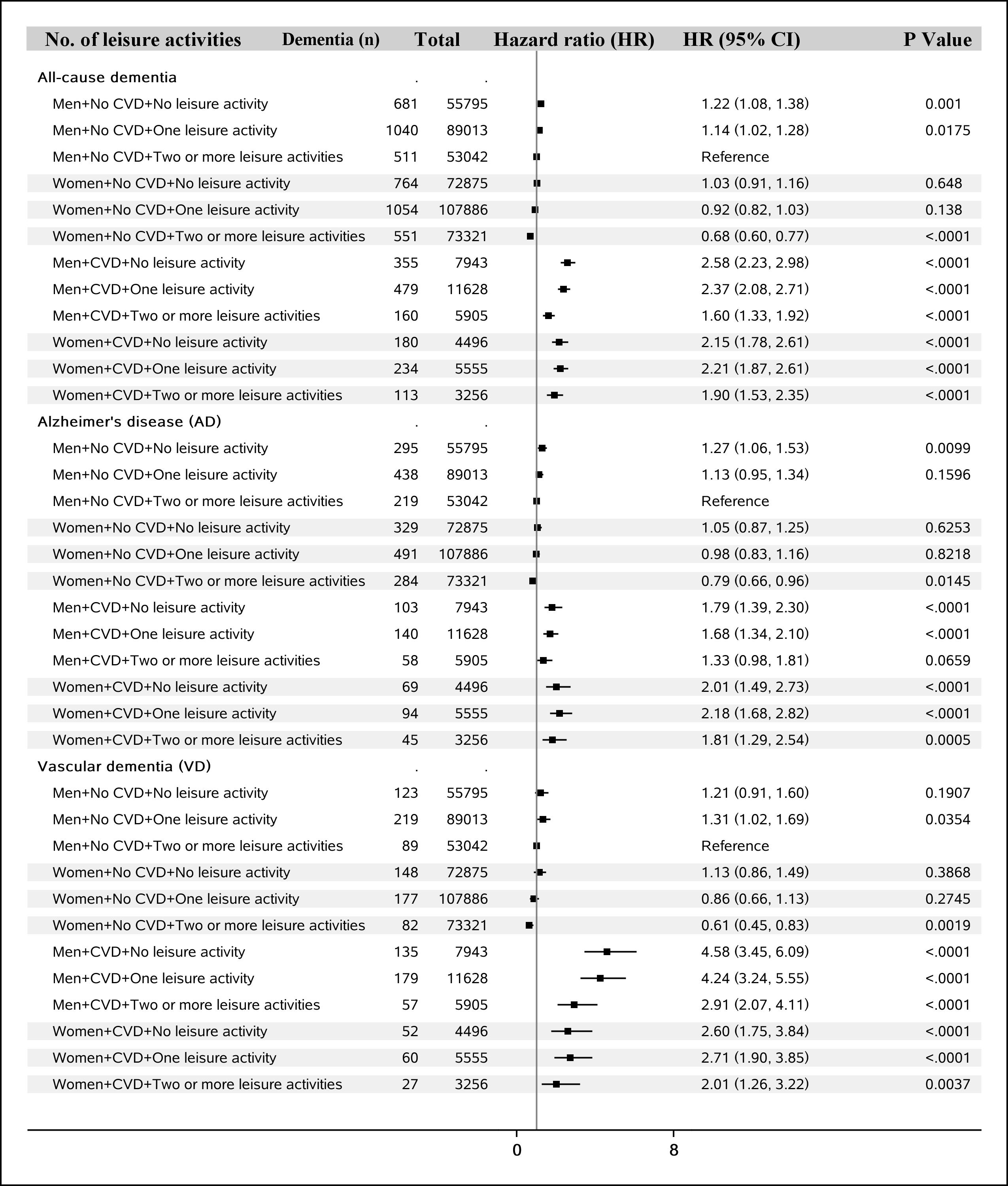


***FIGURE S2:* Sex differences in the association between cardiovascular diseases (CVD) and dementia by number of leisure activities.**

All HRs were adjusted for age at baseline, race/ethnicity, educational years, income level, physical activity level, leisure activities, body mass index (BMI), smoking status, diabetes status, hypertension status and APOE.


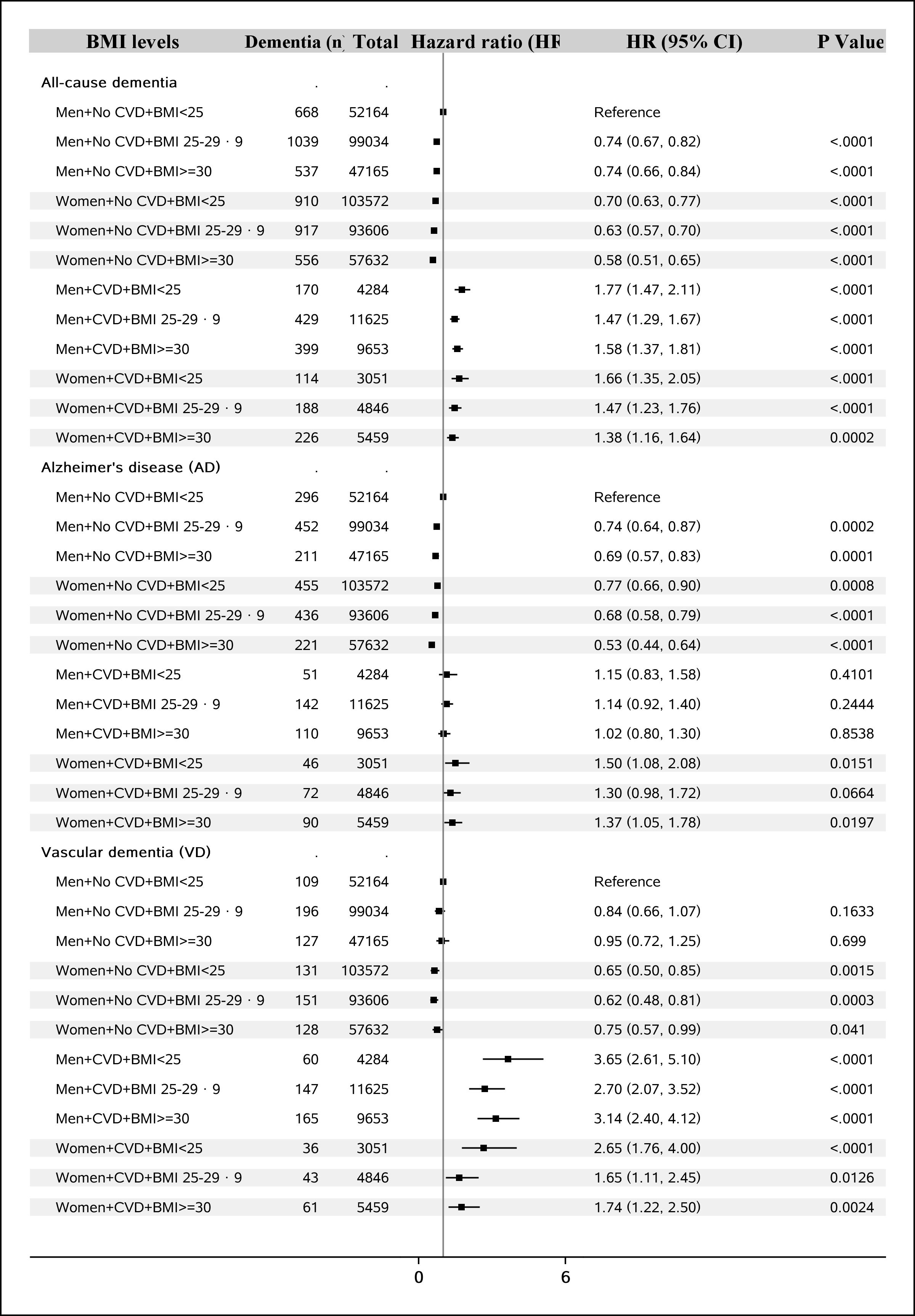


***FIGURE S3:* Sex differences in the association between cardiovascular diseases (CVD) and dementia by body mass index (BMI).**

All HRs were adjusted for age at baseline, race/ethnicity, educational years, income level, physical activity level, leisure activities, body mass index (BMI), smoking status, diabetes status, hypertension status and APOE.


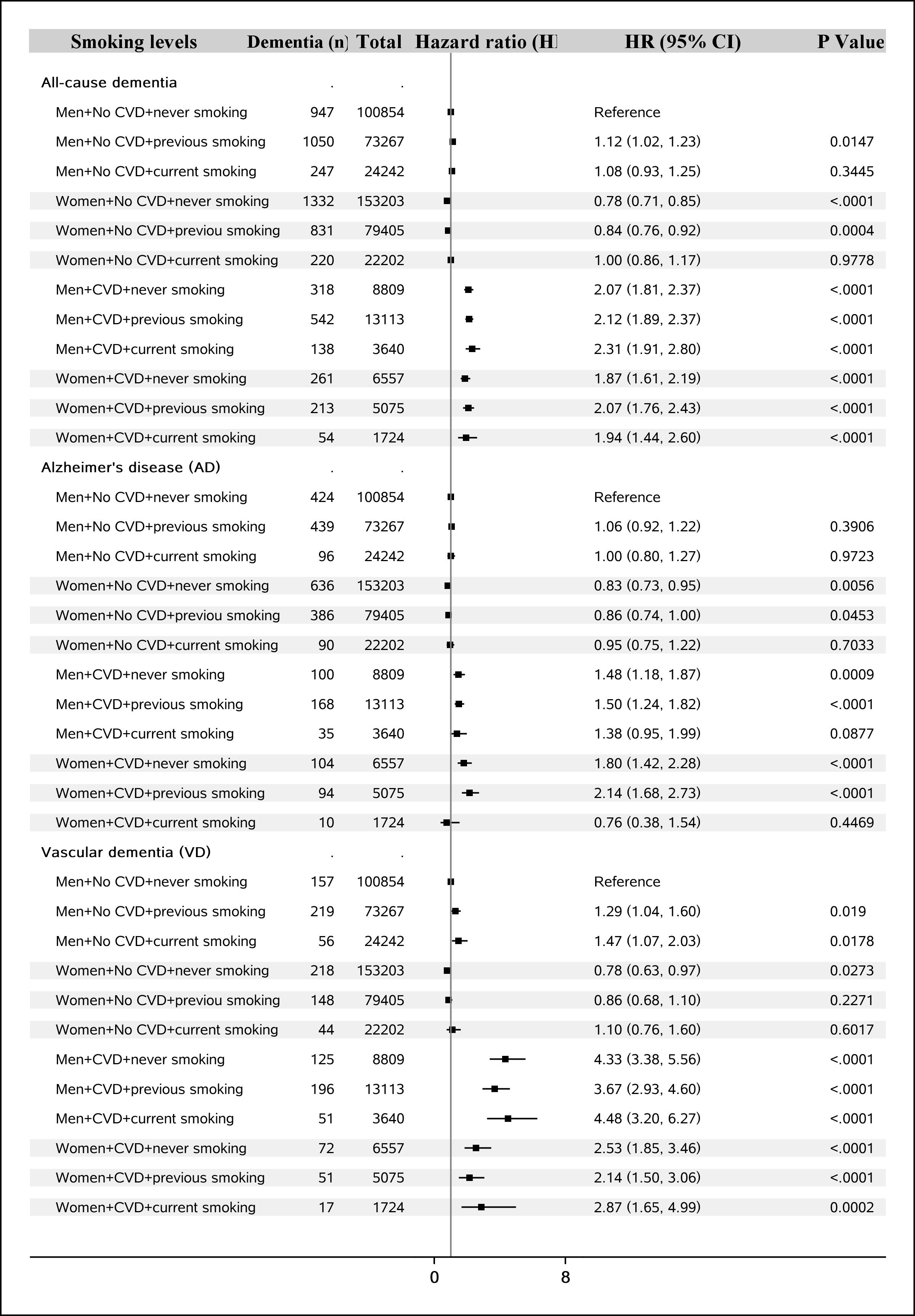


***FIGURE S4:* Sex differences in the association between cardiovascular diseases (CVD) and dementia by smoking status.**

All HRs were adjusted for age at baseline, race/ethnicity, educational years, income level, physical activity level, leisure activities, body mass index (BMI), smoking status, diabetes status, hypertension status and APOE.


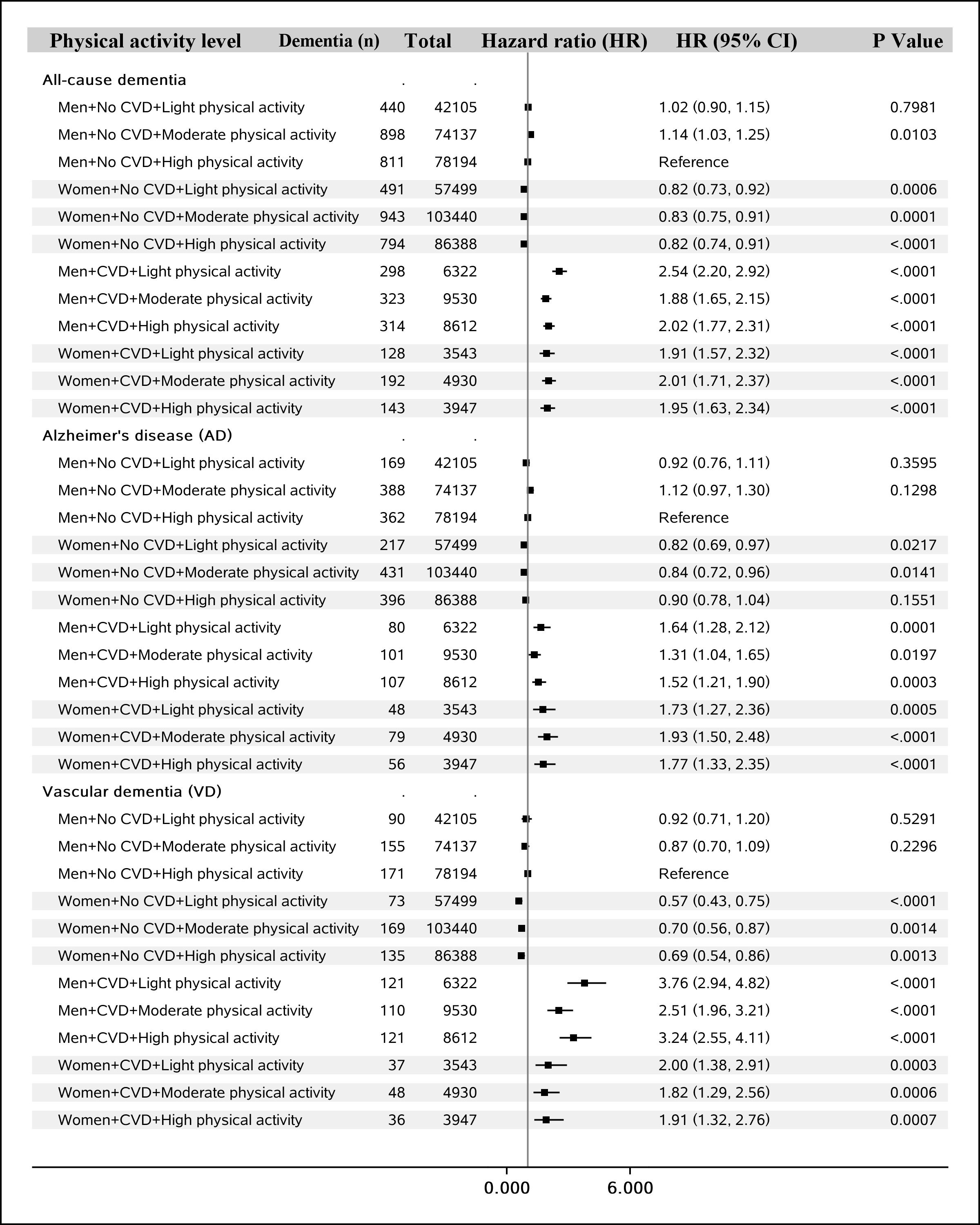


***FIGURE S5:* Sex differences in the association between cardiovascular diseases (CVD) and dementia by physical activities.**

All HRs were adjusted for age at baseline, race/ethnicity, educational years, income level, physical activity level, leisure activities, body mass index (BMI), smoking status, diabetes status, hypertension status and APOE.


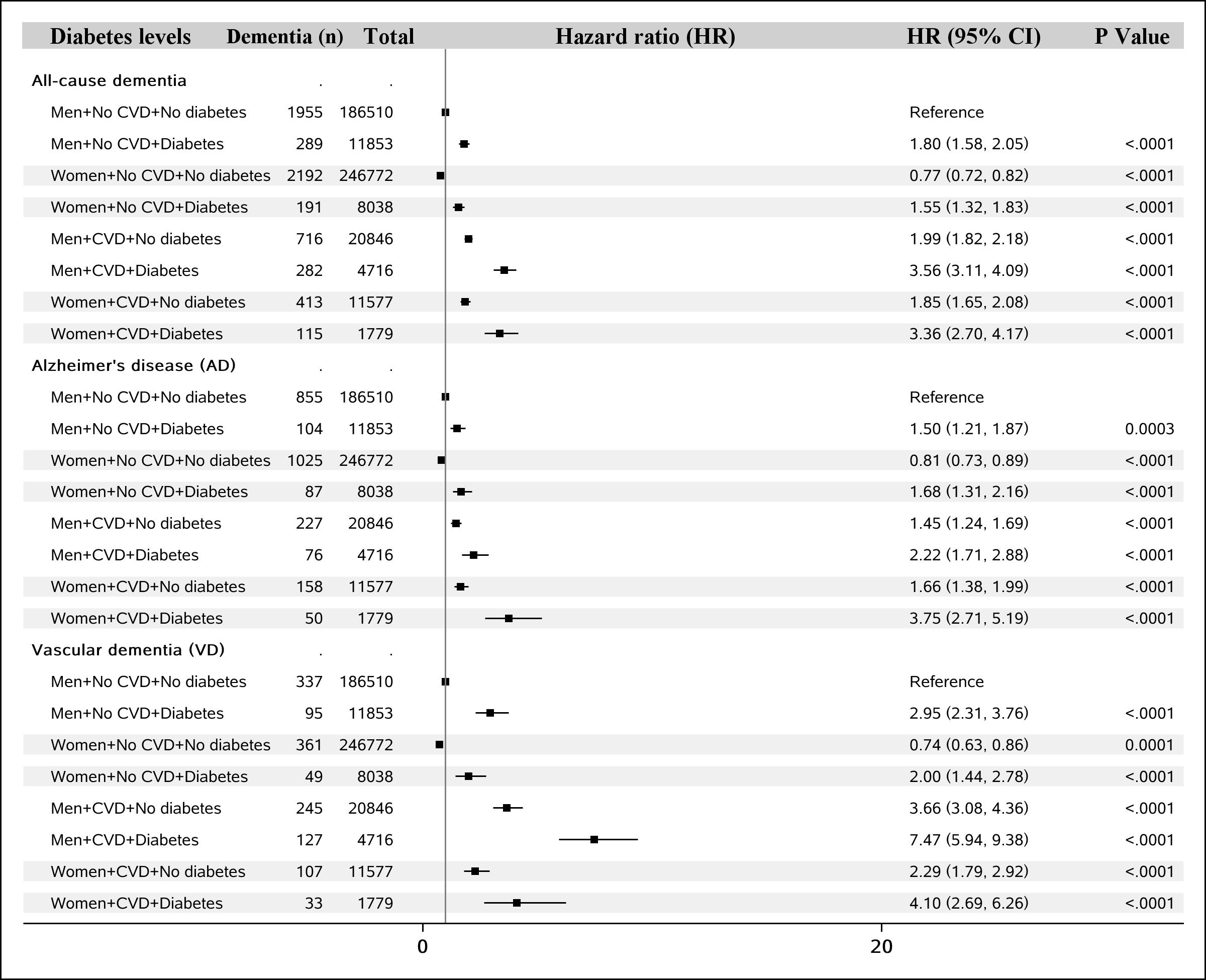


***FIGURE S6:* Sex differences in the association between cardiovascular diseases (CVD) and dementia by diabetes status.**

All HRs were adjusted for age at baseline, race/ethnicity, educational years, income level, physical activity level, leisure activities, body mass index (BMI), smoking status, diabetes status, hypertension status and APOE.


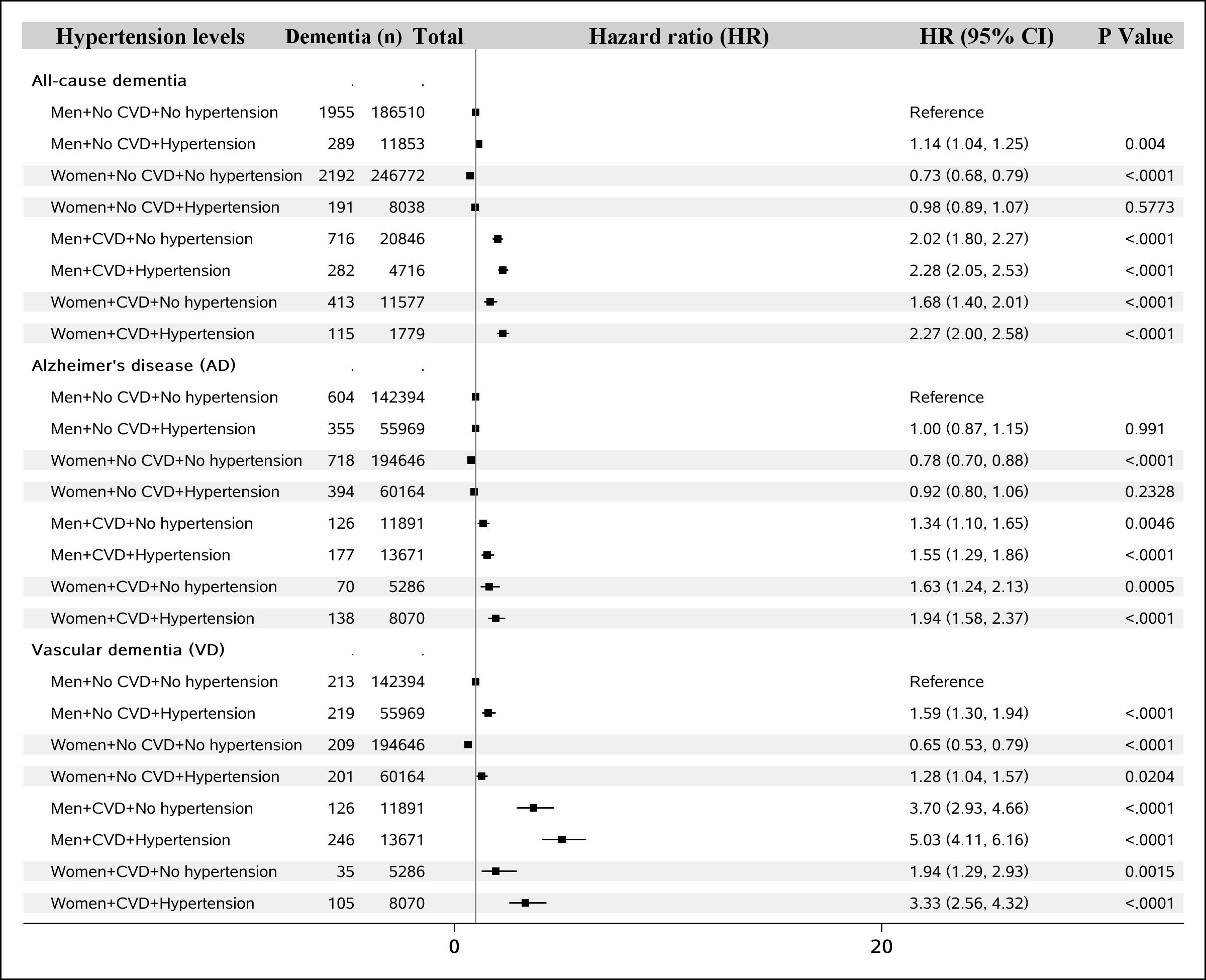


***FIGURE S7:* Sex differences in the association between cardiovascular diseases (CVD) and dementia by hypertension status.**

All HRs were adjusted for age at baseline, race/ethnicity, educational years, income level, physical activity level, leisure activities, body mass index (BMI), smoking status, diabetes status, hypertension status and APOE.
